# Continental-scale patterns of pathogen prevalence: a case study on the corncrake

**DOI:** 10.1111/eva.12192

**Published:** 2014-09-03

**Authors:** Yoan Fourcade, Oskars Keišs, David S Richardson, Jean Secondi

**Affiliations:** 1 Université d'Angers, GECCOAngers, France; 2 Centre for Ecology, Evolution and Conservation, School of Biological Sciences, University of East AngliaNorwich, UK; 3 Laboratory of Ornithology, Institute of Biology, University of LatviaSalaspils, Latvia

**Keywords:** agriculture intensity, approximate Bayesian computation, avian malaria, bird, corncrake, *Crex crex*, effective population size, haemosporidian parasites, parasite transmission

## Abstract

Pathogen infections can represent a substantial threat to wild populations, especially those already limited in size. To determine how much variation in the pathogens observed among fragmented populations is caused by ecological factors, one needs to examine systems where host genetic diversity is consistent among the populations, thus controlling for any potentially confounding genetic effects. Here, we report geographic variation in haemosporidian infection among European populations of corncrake. This species now occurs in fragmented populations, but there is little genetic structure and equally high levels of genetic diversity among these populations. We observed a longitudinal gradient of prevalence from western to Eastern Europe negatively correlated with national agricultural yield, but positively correlated with corncrake census population sizes when only the most widespread lineage is considered. This likely reveals a possible impact of local agriculture intensity, which reduced host population densities in Western Europe and, potentially, insect vector abundance, thus reducing the transmission of pathogens. We conclude that in the corncrake system, where metapopulation dynamics resulted in variations in local census population sizes, but not in the genetic impoverishment of these populations, anthropogenic activity has led to a reduction in host populations and pathogen prevalence.

## Introduction

Pathogens affect host fitness in various ways, including through loss of fecundity and reductions in survival (Lanciani 1975; Smith et al. 2009), and are thus a major driver of evolutionary dynamics (Altizer et al. 2003). The deleterious effects of pathogens can also be a serious threat to any population (McCallum and Dobson 1995; Pounds et al. 2006; Martel et al. 2013), but especially to small populations that already experience elevated extinction risk due to demographic and genetic processes (Saccheri et al. 1998; Bijlsma 2000; O'Grady et al. 2006; Wright et al. 2007). For example, extinction probability is negatively related to population size because of the increasing impact of stochastic environmental events and epizootic infections with decreasing size (Lande 1988). Understanding what factors determine pathogen prevalence is therefore also important to conservation biology (Daszak et al. 2000).

Various ecological parameters influence pathogen infection (Morgenstern 1982; Schrag and Wiener 1995; Plowright et al. 2008). Density-dependent transmission (Dietz 1988; McCallum et al. 2001) has been shown to be responsible for pathogen dynamics in a vast range of host species (see for example, Burdon and Chilvers 1982; Jaffee et al. 1992; Ebert et al. 2000; Hochachka and Dhondt 2000). Together with the density of hosts, the density of vectors may determine infection probability of vector-transmitted pathogens (Trape et al. 1992; Pinto et al. 2000; Sol et al. 2000). Likewise, habitat fragmentation affects pathogen transmission (McCallum and Dobson 2002; Horan et al. 2008), as pathogens spread more rapidly between well-connected habitat patches. Therefore, we may expect habitat quality (driving local carrying capacity) and habitat connectivity (driving colonization/extinction rate and dispersal between populations) to determine the density of hosts and/or vectors. As a consequence, these factors would influence the rate of pathogen transmission within and among populations and, therefore, pathogen prevalence.

Host genetic characteristics also contribute to variation in pathogen distribution across a species range (Frankham et al. 2002; Hawley et al. 2005). Small host populations with depleted genetic diversity appear to be particularly susceptible to pathogens (Spielman et al. 2004) as a result of various genetic factors, including the loss of individual heterozygote advantage (MacDougall-Shackleton et al. 2005; Evans and Neff 2009) and/or the lack of specific alleles conferring resistance within the population level (Hedrick 2002). A negative relationship between host genetic diversity and prevalence is expected if prevalence reliably reflects (*i.e*. is positively correlated to) susceptibility. However, the opposite pattern may be observed if only genetically diverse individual survive infection. So, although it is difficult to determine, *a priori*, the most likely pattern of correlation between pathogen susceptibility and observed infection, it is clear that host genetic diversity – at the population or individual level – can be an important driver of pathogen infection dynamics (Hedrick 2002; Altizer et al. 2003).

Understanding the relative contribution of genetic and ecological factors as drivers of pathogen distribution is a challenging issue. Range-scale studies offer the opportunity to analyse variation in pathogen infection across gradients of ecological conditions and host genetic diversity. However, species with high dispersal capacity and low genetic structuring will provide particularly good systems, in which to investigate the effect of ecological factors on pathogen prevalence, as gene flow will homogenize genetic diversity across their range, thus controlling for the potentially confounding effects of host genetic factors.

Avian malaria, here defined as infection by *Plasmodium* or related genera *Haemoproteus* and *Leucocytozoon* protozoans (Martinsen et al. 2008), has been shown to impact individual survival (Beier et al. 1981; La Puente et al. 2010) and reproductive success (Kilpatrick et al. 2006a; Knowles et al. 2010). Such haemosporidian parasites infect almost all bird species ever tested (Valkiūnas 2005), with various levels of pathogen–host specificity (Bensch et al. 2000; Cumming et al. 2013). Parasites of the genera *Plasmodium* and *Haemoproteus* are transmitted via mosquitoes belonging to the family *Culicidae*, while *Leucocytozoon*'s vectors are mainly flies of the family *Simuliidae* (Valkiūnas 2005). The transmission of avian haemosporidian parasites is mostly thought to occur during spring and summer in temperate climates (Atkinson 2008), but can also occur in tropical climates, such as the African wintering grounds of migrant bird species (Loiseau et al. 2012). Molecular methods now allow the rapid and efficient screening of these infections, as well as the identification of the parasite lineages involved (Bensch et al. 2000; Hellgren et al. 2004; Waldenström et al. 2004). Thus, avian malaria has become a model of host–parasite interactions and their impact on host evolution, ecology and conservation (Westerdahl et al. 2005; Asghar et al. 2011; Njabo et al. 2011). Various studies have explored the effect of host genetic diversity on haemosporidian infection status in birds (MacDougall-Shackleton et al. 2005; Ortego et al. 2007). Infection patterns have also been linked to ecological factors at a relative fine scale, such as altitude (Marzal and Albayrak 2012), distance to water (Wood et al. 2007), food availability (Knowles et al. 2011), host density (Isaksson et al. 2013; Lachish et al. 2013) or other habitat characteristics (Lachish et al. 2013; Gonzalez-Quevedo et al. 2014). However, the contribution of ecological factors on the variation in haemosporidian prevalence at larger, continental scale has received little attention.

The corncrake (*Crex crex*) is a widely distributed bird species that breeds in grassland habitats from Western Europe to Siberia (Schäffer and Koffijberg 2004). Its conservation status differs greatly across different regions of its range. In the westernmost areas, agriculture intensification has resulted in the degradation of habitat suitability and, consequently, population fragmentation, thus leading to a decreasing gradient in population census size from Eastern to Western Europe (Green and Rayment 1996; Green et al. 1997; Birdlife International 2013; Fourcade et al. 2013). Interestingly, spatial genetic structure is weak across the European range, and gene flow from the eastern to the western sites appears to maintain high genetic diversity in all populations (Y. Fourcade, D. S. Richardson, O. Keišs, M. Budka, R. E. Green, S. Fokin, S. Secondi, unpublished data). This species, as well as many farmland bird species in Europe (Donald et al. 2001), has seen its distribution and population trends shaped by anthropogenic activity during the last century. Such disturbance, occurring over a large geographic scale and an extended period, may have disrupted previous host–parasites dynamics and could thus pose overlooked threats to these already declining populations. Therefore, analysing the current patterns of pathogen infections and their ecological drivers seems essential to efficiently anticipate long-term conservation actions.

Here, we investigated the geographic pattern of haemosporidian infection (as a model of a widespread pathogen), in relation to ecological factors across the corncrake's European breeding range. Infection status, and the identity of infecting parasites lineages, was determined for all individuals across populations using molecular screening (Hellgren et al. 2004). To test whether host genetic diversity influences malaria prevalence despite the very low interpopulation variation in this parameter, we first verified that prevalence was uncorrelated with estimates of genetic diversity calculated using a suite of microsatellite markers. Second, we tested the effects of various ecological factors, including climate, host population size (census compared with effective population size) and mean agricultural yields on malaria prevalence. We discuss the implications our results have in regard to understanding the large-scale structuring of pathogen faunas within animal populations and, more specifically, what implications this may have for corncrake conservation.

## Material and methods

### Study species and sample collection

The corncrake (*Crex crex*) is a migratory bird that breeds in the Palearctic, from Western Europe to Baikal Lake, and winters in southeast Africa. On its breeding ground, it occurs mainly in natural or semi-natural grasslands such as floodplain meadows, alpine grasslands or steppes (Schäffer and Koffijberg 2004). We sampled nine European populations (Table1) following the longitudinal demographic gradient that occurs in Europe. Blood samples from 354 corncrakes were collected in 2011 and 2012 during the peak breeding period (May–July). Between 11 pm and 3 am birds were attracted using playback of conspecific male calls and captured with a dipnet or by hand. This method captures males only. Small (ca. 25 μL) blood samples were collected from the brachial vein and stored in absolute ethanol. Each bird was ringed before being released to avoid resampling the same individual within or between years.

**Table 1:** Number of infected corncrakes and prevalence per haemosporidian lineage, for the nine sampling sites across Europe. Sampling sites are ordered from west to east. GenBank accession numbers are provided behind each lineage name.

Location	Long	Lat	Sample size	Infected (prevalence)	Number of positive infections per haemosporidian lineage per population (prevalence)									
					ACCTAC01*	SYBOR10*	WA42*	RTSR1*	SW2*	CRECRE1*	SW5*	WW2†	SYBOR08‡	CIAE02‡
					*EU810700*	*DQ368390*	*EU810615*	*AF495568*	*AF495572*	*KJ783457*	*AF495574*	*AY831755*	*DQ847239*	*EF607287*
France	−0.51	47.58	60	2 (0.03)	2 (0.03)									
Germany	14.30	53.05	34	0 (0.00)										
Czech Republic	16.49	50.24	24	3 (0.13)		1 (0.04)	1 (0.04)	1 (0.04)						
Poland [north]	20.40	54.31	45	7 (0.16)					6 (0.13)	2 (0.04)		1 (0.02)		1 (0.02)
Poland [south]	22.06	49.29	33	4 (0.12)					4 (0.12)					
Poland [east]	23.23	52.59	34	5 (0.15)		1 (0.03)			4 (0.12)	2 (0.06)				
Latvia	23.67	56.71	71	4 (0.06)					4 (0.06)					
Belarus	24.73	52.66	33	5 (0.15)					4 (0.12)				1 (0.03)	
Russia	39.16	55.87	20	6 (0.30)					3 (0.15)		2 (0.10)			1 (0.05)
Total (mean prevalence)				36 (0.10)	2 (0.01)	1 (0.00)	1 (0.00)	25 (0.07)	4 (0.01)	2 (0.01)	1 (0.00)	1 (0.00)	2 (0.01)	

* *Plasmodium*.

† *Haemoproteus*.

‡ *Leucocytozoon*.

### Haemosporidian parasites screening

DNA was first extracted following a salt extraction protocol (Richardson et al. 2001). Haemosporidian infection was detected using a nested PCR (Hellgren et al. 2004; Waldenström et al. 2004). A first PCR amplifies a 570-bp fragment of the cytochrome *b* gene of species belonging to the genera *Plasmodium*, *Haemoproteus* and *Leucocytozoon*, using the primers HaemNF1 and HaemNR3 (Hellgren et al. 2004). Two different PCRs were then run on an aliquot of the first reaction to amplify a shorter fragment of DNA within the first amplicon. The primers HaemF and HaemR2 (Bensch et al. 2000) were used to amplify a 477-bp fragment of *Haemoproteus* or *Plasmodium*, while the primers HaemFL and HaemR2L (Hellgren et al. 2004) were used to amplify a-475 bp fragment of *Leucocytozoon*s.

The first PCR was run in a volume of 10 μL containing 1 μL of extracted DNA (approximately 10 ng/μL), 5 μL of Qiagen TopTaq, 0.4 μL of each primer (initial concentration: 10 mm) and 3.2 μL of pure water. The reaction was performed according to the following conditions: after incubation at 96°C during 3 min, 20 cycles of 20 s at 94°C, 30 s at 50°C and 45 s at 72°C, following by a final incubation at 72°C for 10 min and 20°C for 5 min. The second reaction used 1 μL of PCR product from the first reaction, with the same proportion of reagents. The first and final incubations were similar to the first PCR, but the cyclic reaction was as follows: 40 cycles of 30 s at 94°C, 45 s at 49°C with *Plasmodium*/*Haemoproteus* primers, or 57°C with *Leucocytozoon* primers, and 45 s at 72°C. The final amplification was visualized on a 2% agarose gel using ethidium bromide to identify infected birds. Positive and negative controls (using either a known infected sample from another bird species or 1 μL H_2_O, respectively) were included in all PCR reactions and on the agarose plates. Each sample was run twice to ensure the detection of infected birds and reduce false negatives. When there was inconsistency between two runs, a third screening was run to ensure the correct assignment of infection status. Only individuals that gave positive results in two runs were counted as being infected.

All positive PCR products were sequenced on an ABI 3730 XL sequencer. Sequences were aligned using BioEdit (Hall 1999) and ClustalW (Thompson et al. 1994). We compared the sequences to homologous sequences deposited in the National Centre for Biotechnology Information (NCBI) GenBank (Benson et al. 2005) and MalAvi (Bensch et al. 2009) databases to identify already known lineages. Exact matches with already published sequences were labelled according to the name of the known strain. When a sequence was already referred to by different names, we chose to keep the first published name. Sequences that differed by 1 bp or more were assigned a new name following the guidelines suggested by Bensch et al. (2009): the abbreviated scientific name of the host species (here CRECRE) followed by a number. The phylogenetic relationships between lineages is given in Figure S1, following the protocol described in Appendix S1.

### Microsatellite genotyping, genetic diversity and effective population size

Each DNA sample was genotyped at 15 microsatellite loci. Eight highly polymorphic markers had been specifically designed for corncrake: *Crex1*, *Crex2*, *Crex6*, *Crex7*, *Crex8*, *Crex9*, *Crex11* and *Crex12* (Gautschi et al. 2002), whereas the other markers were identified as being conserved across a large range of bird species: *CAM18* (Dawson et al. 2013), *TG02-120*, *TG04-12*, *TG04-12a*, *TG04-41*, *TG05-30* and *TG012-15* (Dawson et al. 2010). Full details of the genotyping method and genetic statistics of the markers are given in Y. Fourcade, D. S. Richardson, O. Keišs, M. Budka, R. E. Green, S. Fokin, S. Secondi (unpublished data) and Table S1.

We computed three common estimates of individual multilocus heterozygosity, using ‘Rhh’ R package (Alho et al. 2010): the standardized heterozygosity *stH* (Coltman et al. 1999), the internal relatedness *Ir* (Amos et al. 2001) and the homozygosity by locus *Hl* index (Aparicio et al. 2006). We also estimated population-level heterozygosity and genetic diversity using the following measures, computed with ‘HIERFSTAT’ R package (Goudet 2005): observed heterozygosity *Ho*, gene diversity or expected heterozygosity *He*, rarefied allelic richness *Ar* and the inbreeding coefficient *F*_IS_. The effective population size (*N*_*e*_) was calculated for each sampling site using an approximate Bayesian computation (ABC) (Beaumont et al. 2002) approach. We used simulations already computed to investigate the demographic history of corncrake across Europe (Y. Fourcade, D. S. Richardson, O. Keišs, M. Budka, R. E. Green, S. Fokin, S. Secondi, unpublished data) using the framework implemented in the ‘abc’ R package (Csilléry et al. 2012). The full details of *N*_*e*_ calculation are given in Appendix S2.

### Statistical analyses

We assessed the effect of individual measures of genetic diversity on infection probability using binomial regressions. We computed generalized linear mixed models (GLMMs) with population identity as random effect using the ‘lme4’ R package (Bates et al. 2014). We used linear regressions to test the relationships between haemosporidian prevalence and the three measures of population-level genetic diversity: *Ho*, *Ar* and *F*_IS_.

We then investigated the effect of three main categories of ecological factors on the variation of malaria prevalence:

1. Climate: We obtained climatic variables from the WorldClim project (Hijmans et al. 2005), downloaded at a 2.5-arc-min resolution (www.worldclim.org). The original database contained 19 variables but, as some of them were highly redundant, we selected the subset of eight predictors that described the spatio-temporal variations of temperature and rainfall across the study area: the annual mean temperature (Bio1), the maximum temperature of the warmest month (Bio5), the minimum temperature of the coldest month (Bio6), the temperature annual range (Bio7), the annual precipitation (Bio12), the precipitation of the wettest month (Bio13), the precipitation of the driest month (Bio14) and the precipitation seasonality (Bio15). As they remained strongly intercorrelated, we performed a principal component analysis (PCA) on these eight climatic grids and used the first axis, which accounted for 50.2% of the total climatic variation in the study area, as a predictor variable. This component mostly depicted the west–east longitudinal gradient from the oceanic to the continental climate (Figure S1). To take into account fine-scale variability, we extracted the mean climatic value in a 50-km buffer around each sampling site.
2. Agriculture intensity: The mean wheat yields per country (2012 data) were downloaded from FAOSTAT (Food and Agriculture Organization of the United Nations[Bibr b35], http://faostat.fao.org/, accessed on 11/03/2014) and were used as a proxy for the level of agriculture intensification across Europe.
3. Host population size: We included in our analyses two measures of the corncrake population size, (i) inferred by the national census population sizes of corncrake, obtained from Schäffer and Koffijberg (2004), and (ii) the effective population sizes *N*_*e*_ calculated here from genetic data.

Despite the fact that we retained only four potentially informative variables, it is worth noting that they remained correlated (Variance inflation factors VIF: climate: 3.66, census size: 4.73, effective size: 1.23, yield: 5.09). Therefore, after testing for a relationship between each predictor and prevalence using linear regressions, we carried out model selection based on the corrected Akaike information criterion (AICc) (Burnham and Anderson 2002) to determine the variables or combination of variables, that best explained the observed patterns of prevalence. Model selection was carried out using the ‘MuMIn’ R package (Barton 2013). We carried out the analyses described above for all malaria lineages pooled together, and for SW2 alone, the most common and widespread lineage we detected (see Results section). Additionally, we assessed the linear relationship between haemosporidian lineage richness and the four variables included above.

## Results

### Haemosporidian prevalence and distribution of lineages

**Figure 1:**
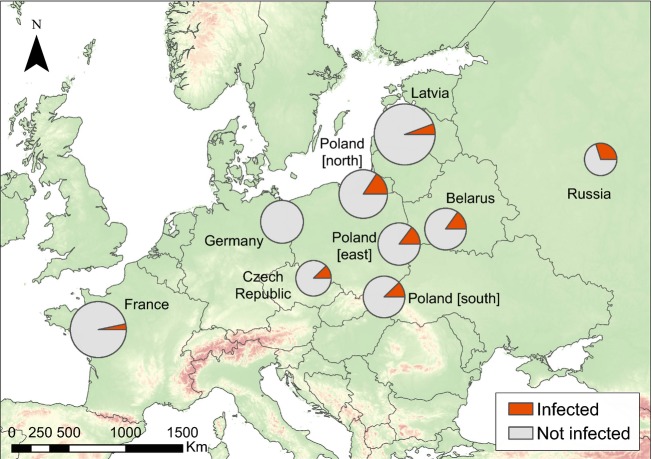
Geographic distribution of malaria prevalence per population across nine European populations of corncrake (*Crex crex*). The size of each circle is function of the number of samples from that location (minimum: Russia, 20 samples; maximum: Latvia, 71 samples).

We found no evidence of cross-sample contamination or failed amplification based on the negative and positive controls. Observed overall prevalence across all populations was 10% (36/354 birds). Prevalence varied considerably among populations across Europe (Range = 0–30%, *χ*² = 18.41, *P *=* *0.018) exhibiting a spatial gradient from south–west (France, 3.3% prevalence) to north–east (Russia, 30% prevalence) (Fig.1, linear regression against longitude: *F*_1,7_ = 13.00, adjusted *R*² = 0.60, *P* = 0.01, linear regression against latitude: *F*_1,7_ = 1.06, adjusted *R*² = 0.01, *P* = 0.34).

Ten different lineages of haemosporidian parasites were detected (Table1): seven *Plasmodium*, two *Leucocytozoon* and one *Haemoproteus* lineage (Figure S2). One bird was found to be infected by both a *Leucocytozoon* strain and a *Plasmodium* strain. Another four Polish birds showed evidence of mixed infection with both the *Plasmodium* strain SW2 and a previously undescribed haplotype that was 1 bp different (CRECRE1; GenBank accession number *KJ783457*). This new lineage was confirmed by the repeated amplification and sequencing of the original DNA sample.

Among the ten haemosporidian strains detected, one *Plasmodium* lineage (SW2) occurred in 71% (25/36) of infected corncrakes (Table1). This haplotype was restricted to the six easternmost populations (Poland, Latvia, Belarus and Russia) with an average prevalence of 11.6% across these locations. SW5 was found only in Russia, infecting two birds. Regarding the western populations, France was characterized by a single lineage found only at this site: ACCTAC01. In the Czech Republic – the westernmost site after France in which haemosporidian parasite was detected – a total of three lineages were found. Two of these lineages, WA42 and RTSR1, occurred only in the Czech Republic, while the lineage SYBOR10 was found here and also in populations further east.

### Relationship between haemosporidian prevalence and genetic diversity

Following a binomial GLMM procedure, we found no effect of standardized heterozygosity (*stH*) on infection probability (Wald *Z* = 0.51, *P* = 0.61). No relationship was detected for the two other predictors either: internal relatedness *Ir* (Wald *Z* = −1.15, *P* = 0.61) and homozygosity by locus *Hl* (Wald *Z* = −1.02, *P* = 0.31). Similarly, we found no effect of genetic estimators of diversity on infection probability when considering only the SW2 lineage (all *P* > 0.5).

Observed heterozygosity (*Ho)* varied between 0.63 and 0.75 among populations, but was not related with haemosporidian prevalence (all lineages: *F*_1,7_ = 0.64, adjusted *R*² = −0.05, *P* = 0.45, SW2: *F*_1,7_ = 0.003, adjusted *R*² = −0.14, *P* = 0.96). Similarly, little variation among populations was observed in allelic richness (*Ar*: 8.95–9.78), gene diversity (*He*: 0.72–0.77) and *F*_IS_ (0.00–0.17), and none of these measures was correlated with haemosporidian prevalence, either for all lineages or for SW2 only (all *P* > 0.1).

### Estimation of effective population size

Overall, the ABC analysis indicated a mean effective population size across all populations of 117 204 ± 65 853 (Table S3, minimum: mode *N*_e_Poland (East)_ = 50 976, 95% CI: 25 787–364 012; maximum: mode *N*_e_Germany_ = 277 179, 95% CI: 123 777–732 928). The estimation of *N*_*e*_ for the whole dataset was higher than for each population separately (mode *N*_e_all-data_ = 385 833, 95% CI: 85 225–744 614) and remained within a plausible range given the estimated European corncrake population size of 2.6–4 million birds (Schäffer and Koffijberg 2004; Birdlife International 2013). *N*_*e*_ did not exhibit any longitudinal or latitudinal pattern (longitude: *F*_1,7_ = 0.003, adjusted *R*² = −0.14, *P* = 0.96; latitude: *F*_1,7_ = 0.0008, adjusted *R*² = −0.14, *P* = 0.98). Census and effective population size estimated per sampling site were not correlated (effective size versus census size: *F*_1,7_ = 0.17, adjusted *R*² = −0.12, *P* = 0.70) (Table S3).

### Relationship between haemosporidian prevalence/richness and ecological factors

**Figure 2:**
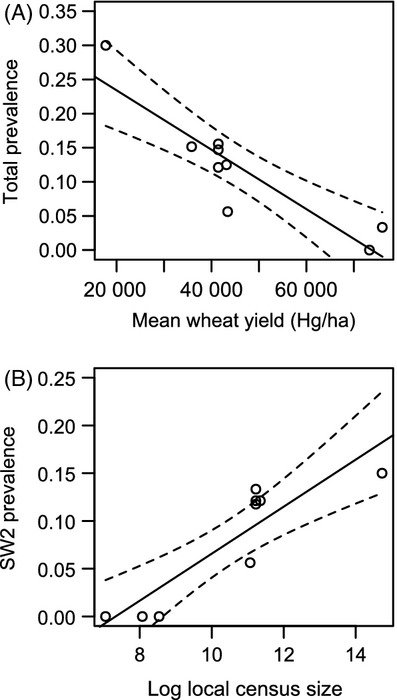
Haemosporidian prevalence in nine European populations of corncrake plotted against (A) agricultural intensity approximated by the mean wheat yield per country (in Hg/ha) for all haemosporidian lineages pooled and (B) corncrake local census population size for the most widespread lineage only (SW2).

We found that total haemosporidian prevalence exhibited a significant negative relationship with climate (*F*_1,7_ = 7.54, adjusted *R*² = 0.45, *P* = 0.03) and a positive relationship with agricultural yield (*F*_1,7_ = 29.91, adjusted *R*² = 0.78, *P* < 0.001) and corncrake census size (*F*_1,7_ = 14.48, adjusted *R*² = 0.63, *P* < 0.001), but not with effective population size (*F*_1,7_ = 0.33, adjusted *R*² = −0.09, *P* = 0.58). Among these variables, the model selection procedure identified agricultural yield as the most important factor influencing total haemosporidian prevalence (Table2 and Fig.2A). All other models greatly departed from this one regarding ΔAICc (difference with 2nd best model = 4.87), showing that the other predictors poorly explained the observed variation of prevalence compared with yield.

**Table 2:** Results of model selection by AICc. Linear models linking haemosporidian infection and ecological predictors, for all lineages and for SW2 lineage only, are ranked by AICc. For visual convenience, only models that had an AICc weight >0.01 are shown. Yield is the mean wheat yield per country as provided by the FAO. The climate variable is a synthetic climatic predictor extracted from a PCA on the *Bioclim* dataset (Hijmans et al. 2005). Census and effective sizes are corncrake population size inferred, respectively, from field surveys (Schäffer and Koffijberg 2004) and genetic analyses.

	Adj. *R*²	*F*	df	AICc	ΔAICc	AICc weight
All lineages						
Yield	0.78	29.91	3	−23.50	0.00	0.83
Census size	0.63	14.48	3	−18.60	4.87	0.07
Yield + Census size	0.75	13.30	4	−16.60	6.93	0.03
Yield + Effective size	0.75	12.96	4	−16.40	7.12	0.02
Yield + Climate	0.75	12.82	4	−16.30	7.20	0.02
Climate	0.45	7.54	3	−15.10	8.39	0.01
SW2						
Census size	0.77	27.33	3	−28.60	0.00	0.69
Census size + Effective size	0.84	21.51	4	−26.00	2.61	0.19
Yield	0.58	11.84	3	−23.30	5.40	0.05
Census size + Climate	0.77	14.77	4	−23.10	5.50	0.04
Census size + Yield	0.73	11.78	4	−21.50	7.16	0.02

Considering only SW2 prevalence, a similar positive relationship was found with corncrake census size (*F*_1,7_ = 27.30, adjusted *R*² = 0.76, *P* = 0.001) and agricultural yield (*F*_1,7_ = 11.84, adjusted *R*² = 0.55, *P* = 0.01), but the regression with the climate principle component was not significant anymore (*F*_1,7_ = 3.84, adjusted *R*² = 0.26, *P* = 0.09). Again, the relationship with corncrake effective population size was not significant (*F*_1,7_ = 1.48, adjusted *R*² = 0.06, *P* = 0.26). However, here, the best model explaining SW2 prevalence included only corncrake population census size (ΔAICc with 2nd best model = 2.61) (Table2 and Fig.2B). In this case, agricultural yield, which was ranked first for total prevalence, appeared only in third position (ΔAICc with best model = 5.40).

No significant linear relationship was identified between lineage richness and the four predictors tested (all *P* > 0.05). The relationship between richness and agricultural yield approached significance though (*F*_1,7_ = 4.79, df = 7, adjusted *R*² = 0.32, *P* = 0.06).

## Discussion

Mean prevalence of haemosporidian infection across the European range of the corncrake was ca. 10%, which is relatively low compared with other bird species. For example, an analysis of blood parasites across 74 passerine species revealed an average prevalence of 26% (Scheuerlein and Ricklefs 2004). Similarly, a 39% prevalence was found among 50 bird species sampled in Dominican Republic (Latta and Ricklefs 2010). However, in the corncrake, haemosporidian prevalence showed a strong geographic gradient, increasing from Western to Eastern Europe. Interestingly, the prevalence of easternmost populations was consistent with the average value given above, whereas western populations appear to be almost free of these parasites. In the corncrake, where individual or population heterozygosity had no effect on haemosporidian infection, prevalence was strongly related with agriculture yield per country. However, when only the most widespread lineage SW2 was considered, the most important factor explaining prevalence was local corncrake census size.

The lack of relationships between haemosporidian prevalence and host genetic diversity is consistent with our predictions. As a consequence of high gene flow, no loss of genetic diversity occurred in the threatened westernmost populations. Indeed, genetic diversity varied little between populations (*H*_*o*_: 0.63–0.75) and the estimates of effective population size provided by the ABC analysis were totally unrelated to the survey-based population estimates. Therefore, corncrake genetic characteristics cannot explain the spatial variation in haemosporidian prevalence. As genetic diversity differs so little between populations, ecological factors must account for the marked spatial variation of haemosporidian prevalence across the corncrake range. A likely explanation is that haemosporidian prevalence is driven by vector density (Trape et al. 1992; Loaiza and Miller 2013). This hypothesis is supported by the negative relationship between haemosporidian prevalence and agricultural yield. Differences of vector density may be caused by variation in natural environmental conditions or in the intensity of human disturbance. The massive drainage of wetlands (Brinson and Malvárez 2002), and intensive use of pesticides in farmland (Geiger et al. 2010) across Western Europe, may have reduced the number of vectors, either by directly reducing vector populations or by indirectly reducing the size of other host bird populations (Donald et al. 2001; Stoate et al. 2009). It has been shown that agriculture intensification can lead to a decline of *Diptera* abundance (Wickramasinghe et al. 2004; Paquette et al. 2013). In contrast, in an island system, Gonzalez-Quevedo et al. (2014) showed that anthropogenic activity, specifically the creation of water reservoirs and poultry farms, can increase avian malarial infection within a natural bird population. In Europe, agricultural practices show a gradient of intensity from west to east which may affect vector fitness and, as a consequence, have generated the gradient of haemosporidian prevalence in corncrake populations that we observe. The reasons for such large-scale variation in the density of malaria vectors have never been investigated. Human-driven changes of the environment operate at the ecosystem scale, and it seems likely that both vector and host densities have experienced the same gradient of alteration in the last decades. Although our results did not provide direct evidence, they appear to support the hypothesis that agricultural intensity has affected pathogen communities.

Although at the scale of the whole haemosporidian community, the intensity of agriculture appeared to be the main driver of prevalence, it is noticeable that, when we focused on a single malaria lineage (here SW2), haemosporidian prevalence was highly correlated with the gradient in host census sizes across Europe. Classically, host density is a key factor that determines parasite transmission (Dietz 1988), including in malaria (Lachish et al. 2011; Isaksson et al. 2013). It could account for the observed variations of prevalence at the scale of the SW2 lineage. In our sampling, most infected birds carried this very generalist haemosporidian lineage. It has been described as *Plasmodium homonucleophilum* (Ilgūnas et al. 2013) and has been identified in numerous bird species, including sedge warbler *Acrocephalus schoenobaenus* (Waldenström et al. 2002), great tit *Parus major* (Beadell et al. 2006) and tawny owl *Strix aluco* (Krone et al. 2008). Therefore, its transmission relies on a range of hosts and does not depend on corncrake only, which at first sight limits the impact that corncrake density alone should have on its prevalence. Nevertheless, the observed gradient of corncrake population size along the gradient of agriculture intensity is likely to exist in many bird species affected by agricultural practices (Donald et al. 2001), so the overall pool of host species may exhibit the same pattern, thus influencing parasite transmission. Moreover, corncrake males tend to aggregate on specific calling sites during the breeding season (Budka and Osiejuk 2013; Ręk 2014) and such behaviour certainly favours density-dependent pathogen transmission. Furthermore, although we do not have direct measures of local density, the large populations of corncrakes in Eastern Europe should result in much higher within-patch local densities or higher densities of such breeding areas, than in Western Europe, both of which would facilitate transmission of haemosporidian parasites. Moreover, the large populations in Eastern Europe may provide a reservoir of chronically infected birds that contributes to the maintenance of relatively high prevalence.

The identity of haemosporidian lineages provides some alternative explanations for the observed pattern. Indeed, most infected birds in Eastern Europe were carriers of SW2, while this lineage was absent from the western sites. This generalist lineage was already identified in several western locations (for example, United Kingdom (Szöllősi et al. 2011) or Portugal (Ventim et al. 2012)) as well as Eastern European countries (for example, Romania (Svoboda et al. 2009) and Russia (Ilgūnas et al. 2013)). Clearly, its range is not restricted to Eastern Europe. Therefore, the low prevalence in western sites may explain why the SW2 lineage was not detected there. Nevertheless, these results raise questions about the geographic structure of haemosporidian lineages across the corncrake range. Its distribution may be explained by the use of alternate migration routes and/or wintering areas (Rintamäki and Ojanen 1998; Wirth et al. 2005; Durrant et al. 2008). There are some data to support this hypothesis. We found evidence that the French and the Scottish population (the latter was not sampled in a way that allowed for disease screening) differ genetically and morphologically from the rest of the Europe corncrakes (Y. Fourcade, D. S. Richardson, O. Keišs, M. Budka, R. E. Green, S. Fokin, S. Secondi, unpublished data). Similarly, recent data about corncrake migration suggest that birds breeding in Britain may use a different migration pathway than more eastern populations (Green 2013). If the French birds also follow this alternative western migration route, and providing the haemosporidian infections are acquired in wintering grounds, this may explain why this population differs so clearly in terms of the genetic identity and prevalence of pathogens found there. However, this issue remains rather speculative and needs further investigation. Indeed, most haemosporidian strains identified here have already been found in migratory hosts, both in Africa and in Europe. For example, ACCTAC01, the *Plasmodium* lineage found in France, has also been identified in resident African species, such as the African Goshawk *Accipiter tachiro*, showing that infection may occur in Africa. In contrast, the widespread SW2 lineage has been found in a nonmigrant European species, the tawny owl *Strix aluco* (Krone et al. 2008), showing that this parasite can be acquired in the corncrake's breeding grounds. Although infections sites are unknown in the present case, the clear longitudinal pattern of prevalence that we observed in Europe suggests that it depends on factors occurring in the breeding area. Furthermore, there is no explanation why processes occurring in winter would determine the relationships between prevalence and agriculture intensity in Europe.

We predicted, and confirmed, that host genetic diversity would not be driving patterns of pathogen prevalence in the corncrake system because gene flow maintains equally high diversity level across the European range. Therefore, the large variation in haemosporidian prevalence observed must be explained by ecological factors. The longitudinal gradient of haemosporidian parasites prevalence correlated with wheat yields, used here as a proxy for agriculture intensity. Focusing on a single lineage, the most important variable driving prevalence was host population size, but again, this factor is directly linked to agriculture activity which contributes to the gradient of corncrake population sizes. A likely explanation is that agriculture intensification in Western Europe has led to reduced infection by strongly limiting both vector and host density. A practical consequence is that infection by haemosporidians – or other pathogens borne by insect vectors and/or where transmission is density dependent – should not be a major threat to the viability of these small bird populations. Our results also suggest that the massive decline of corncrake in Western Europe can be largely imputed to agriculture practices and not to other neglected factors such as pathogens. Thus, efficient conservation actions could be largely inspired by those applied in United Kingdom – based on the management of mowing practices – as they managed to halt the decrease of the species and eventually to recover a significant corncrake population (O'Brien et al. 2006).

As already stated, the areas of low haemosporidian prevalence may indicate a deterioration of grassland ecosystems with an extirpation of most insect vectors or a disruption of parasitic cycles. At the European scale, agricultural intensity has been shown to be linked to a decline of arthropod communities in farmland landscapes (Hendrickx et al. 2007; Le Féon et al. 2010). As a global decrease of insect populations is observed (Dunn 2005; Conrad et al. 2006), managing insect populations is becoming a major issue because their decline directly affects ecosystem services such as pollination (Potts et al. 2010). Therefore, efforts should be made to implement conservation strategies that maintain both biodiversity and functional relationships like host–parasite interactions. In this regard, parasites screening in birds hosts may serve in monitoring insect populations and functional interactions and may thus provide wider insights into biodiversity conservation in agricultural landscapes. More generally, our study system allowed us to assess the effect of large-scale ecological factors on prevalence patterns. Further continental-wide studies are needed that provide insights about the relative contribution of extrinsic (ecological) and intrinsic (genetic) factors on pathogen prevalence. These may not only provide ecological and evolutionary understanding of pathogen dynamics, but may also improve the design of conservation strategies for wild populations potentially threatened by pathogens (De Castro and Bolker 2004; Smith et al. 2009). They may also help to predict the spread of zoonotic diseases carried by migrating animals (see examples for avian influenza (Reed and Meece 2003; Gilbert et al. 2006; Kilpatrick et al. 2006b)).

## Data archiving statement

Data for this study are available at: New *Palsmodium* sequence (CRECRE1): GenBank accession number KJ783457. Infection status of individuals: Dryad Digital Repository: http://doi.org/10.5061/dryad.gt86f.

## Supporting Information

**Appendix S1.** Phylogenetic analyses of malaria lineages detected in populations of the corncrake. **Appendix S2.** Method used for the estimation of effective population size by ABC. **Table S1.** Basic statistics for each microsatellite locus. **Table S2.** Posterior probability of demographic models inferred by ABC. **Table S3.** Estimates of effective population sizes and local census population sizes. **Figure S1.** Synthetic climatic predictor, obtained from the first axis of a PCA performed on a set of eight bioclimatic variables. **Figure S2.** Phylogenetic tree of the ten malaria lineages detected.
